# Extreme Biocatalysts: From Basic Research to Biotechnological Applications

**DOI:** 10.3390/ijms25126492

**Published:** 2024-06-12

**Authors:** Salvatore Fusco, Bettina Siebers

**Affiliations:** 1 Biochemistry and Industrial Biotechnology (BIB) Laboratory, Department of Biotechnology, University of Verona, 37134 Verona, Italy; 2 Molecular Enzyme Technology and Biochemistry, Environmental Microbiology and Biotechnology, Centre for Water and Environmental Research, University of Duisburg-Essen, 45117 Essen, Germany; bettina.siebers@uni-due.de

Biocatalysis, a cornerstone of modern biotechnology, is poised to revolutionize industrial processes across diverse sectors [1]. Its potential to drive sustainable practices aligns seamlessly with the urgent global shift toward a circular economy [2]. Despite this promise, the comprehensive utilization of biocatalysts has been hindered by their limited stability under harsh industrial conditions [3]. In this context, extremophiles and their enzymes offer a treasure trove of solutions to overcome these limitations [4,5]. In the following Special Issue, we embark on a journey into the realm of extreme biocatalysis, exploring the unique properties of extremozymes and their applications in diverse biotechnological contexts.

For instance, the utilization of lignocellulosic biomasses presents both environmental challenges and opportunities for sustainable biofuel and chemical production. In one manuscript included in the present Special Issue, researchers investigated the cultivation of microbial communities on spent mushroom substrate (SMS), a renewable resource abundant in cellulose and hemicellulose. Through metagenomic analysis, they uncovered the temperature-dependent preferences of microbial communities for cellulose, hemicellulose, and lignin degradation. Notably, thermophilic microbiomes secrete robust lignocellulolytic enzymes, showcasing their potential for industrial biorefinery applications (Contribution 1). The above findings underscore the importance of tailored strategies for harnessing microbial biotechnological potential in biomass conversion processes.

Aldehyde:ferredoxin oxidoreductases (AORs) play a crucial role in anaerobic metabolism, catalysing the oxidation of aldehydes to acids using ferredoxin as a cofactor [6]. Undertaking a biochemical characterization of an AOR from the thermophilic bacterium *Thermoanaerobacter* sp., researchers unveiled its broad substrate specificity and remarkable stability across a wide range of temperature and pH conditions (Contribution 2). The above study lays the groundwork for future investigations into the structure–function relationships of AORs and their potential applications in biotechnological processes.

Red algae and their associated bacteria constitute efficient metabolic cycling systems in marine ecosystems [7]. Through metagenomic analysis, researchers dissected the microbial diversity and enzymatic repertoire of red algae-associated bacteria across diverse geographical regions. They uncovered significant differences in bacterial community composition and enzyme abundance, shedding light on the complex interplay between environmental factors and microbial ecology (Contribution 3). Such insights deepen our understanding of algal microbiome dynamics and hold implications for biotechnological applications in biomass degradation and environmental remediation.

Thermophilic L-asparaginases (L-ASNases) are key therapeutic enzymes used in cancer treatment [8,9]. In a study aiming to improve their catalytic efficiency, researchers employed rational protein engineering strategies to enhance the activity of a thermophilic L-ASNase from *Thermococcus sibiricus*. Through targeted mutations adjacent to the substrate-binding site, they achieved a significant increase in enzyme activity and cytotoxicity against cancer cell lines (Contribution 4). The above work highlights the potential of protein engineering in enhancing the efficacy of therapeutic enzymes for biomedical applications.

Nucleoside analogues hold immense therapeutic potential for treating viral infections and different types of cancer [10,11]. In their study, Thiele et al. demonstrated the feasibility of continuous nucleoside synthesis using enzyme membrane reactors (EMRs). By immobilizing enzymes within a membrane, they achieved high productivity and cost-efficiency rates in nucleoside production, surpassing those of traditional batch reactions (Contribution 5). The above technology offers a sustainable alternative for nucleoside synthesis, with implications for pharmaceutical manufacturing and biocatalyst optimization.

Thermophilic microorganisms, adapted to thrive at high temperatures, harbour a rich reservoir of extremozymes with industrial applications [12]. Through functional genomic annotation, researchers identified a novel glycosyl hydrolase from *Alicyclobacillus mali* FL18, exhibiting broad substrate specificity and optimal activity at elevated temperatures (Contribution 6). This enzyme holds promise for lignocellulose deconstruction and biofuel production, highlighting the biotechnological potential of thermophilic microorganisms in sustainable industrial processes [13].

Xylanases play a crucial role in enhancing pulp bleachability in the paper industry; however, their industrial implementation faces challenges due to harsh alkaline conditions [14,15]. In one of the studies included in this Special Issue, researchers engineered a highly thermo- and alkaline-tolerant xylanase from *Pseudothermotoga thermarum*, enabling significant chlorine dioxide savings in elemental chlorine-free (ECF) bleaching processes (Contribution 7). This enzymatic approach offers a sustainable solution for reducing environmental impact while maintaining pulp quality and process efficiency.

Two research papers included in this Special Issue focused on metagenomic analyses, and their findings revealed: (i) a diverse microbial community in the Muiño da Veiga hot spring in Spain, including novel enzymes and microorganisms with potential biotechnological applications (Contribution 8), and (ii) a new short-chain dehydrogenase/reductase (SDR) from an Icelandic hot spring metagenome (Contribution 9). This enzyme demonstrated versatility in reducing various carbonylic substrates and showed high thermostability and solvent tolerance, highlighting its synthetic application potential.

In addition to original research articles, the following Special Issue includes insightful reviews that provide comprehensive overviews of cold-active enzymes for industrial applications (Contribution 10) and the biotechnological potential of halophilic filamentous fungi (Contribution 11). These reviews not only synthesize existing knowledge but also offer valuable insights into future research directions, highlighting the importance of interdisciplinary collaboration in advancing biocatalysis research.

In conclusion, the manuscripts featured in this Special Issue illuminate the vast potential of extreme biocatalysis in driving sustainable industrial processes and addressing global challenges. From lignocellulosic biomass conversion to pharmaceutical synthesis and pulp bleaching, extremozymes offer versatile solutions across diverse sectors. As we continue to unlock the secrets of extremophiles and their enzymes, let us seize the opportunity to harness their biotechnological potential for a more sustainable future.
